# Changes in Lipidomics, Metabolomics, and the Gut Microbiota in CDAA-Induced NAFLD Mice after Polyene Phosphatidylcholine Treatment

**DOI:** 10.3390/ijms24021502

**Published:** 2023-01-12

**Authors:** Jiayuan Zhang, Xiaoling Zang, Jinxiao Lv, Yicong Zhang, Zhihua Lv, Mingming Yu

**Affiliations:** 1 School of Medicine and Pharmacy, Ocean University of China, Qingdao 266003, China; 2 Laboratory for Marine Drugs and Bioproducts of Qingdao National Laboratory for Marine Science and Technology, Qingdao 266003, China

**Keywords:** NAFLD, CDAA-induced, lipidomics, metabolomics, gut microbiota

## Abstract

Non-alcoholic fatty liver disease (NAFLD) is the most common chronic liver disease in most parts of the world. Although there is no first-line drug approved for the treatment of NAFLD, polyene phosphatidylcholine (PPC) is used by clinicians to treat NAFLD patients. This study aimed to evaluate the efficacy of PPC on a mice model of NAFLD, and to study the PPC’s mechanism of action. The mice were fed a choline-deficient, L-amino acid-defined (CDAA) diet to induce NAFLD and were subsequently treated with PPC. The treatment effects were evaluated by the liver index, histopathological examination, and routine blood chemistry analyses. Lipidomics and metabolomics analyses of 54 samples were carried out using ultraperformance liquid chromatography (UPLC) coupled to a mass spectrometer to select for changes in metabolites associated with CDAA diet-induced NAFLD and the effects of PPC treatment. The intestinal flora of mice were extracted for gene sequencing to find differences before and after the induction of NAFLD and PPC treatment. PPC significantly improved the CDAA diet-induced NAFLD condition in mice. A total of 19 metabolites including 5 polar metabolites and 14 lipids showed marked changes. In addition, significant differences in the abundance of *Lactobacillus* were associated with NAFLD. We inferred that the protective therapeutic effect of PPC on the liver was related to the supplement of phosphatidylcholine, lysophosphatidylcholine, and sphingomyelin (PC, LPC, and SM, resectively) and acylcarnitine metabolism. This study developed a methodology for exploring the pathogenesis of NAFLD and can be extended to other therapeutic agents for treating NAFLD.

## 1. Introduction

Nonalcoholic fatty liver disease (NAFLD) is a metabolic stress-related liver disease defined as the hepatic accumulation of lipids, mainly triglyceride, in the absence of substantial alcohol consumption (<20 g/day) or other secondary causes [1,2]. An important characteristic of nonalcoholic steatosis is the accumulation of triglyceride (TG) and total cholesterol (TC) in hepatocytes. Some patients with nonalcoholic fatty liver disease develop NASH and fibrosis, increasing the risk of cirrhosis and even hepatocellular carcinoma [3]. The pathophysiology of NAFLD has not been completely elucidated [4]. Currently, the “multiple hit” is one of the most widely accepted models to explain the progression of NAFLD. The “multiple hit” hypothesis considers multiple insults acting together on genetically predisposed subjects to induce NAFLD. Such hits include insulin resistance, hormonal secretion from the adipose tissue, nutritional factors, and the gut microbiota, as well as genetic and epigenetic factors [5]. Recent studies show that the gut microbiota is an important factor that should be taken into consideration when studying NAFLD [6].

As there is no first-line drug for NAFLD, clinicians use polyene phosphatidylcholine (PPC) to treat these patients [7]. The main component of PPC is extracted from the soybean. The therapeutic and protective effects of PPC on the liver have been reported in many studies [8,9]. However, there have been few systematic studies on its mechanisms of action, and this study aims to explore the mechanisms of PPC action on non-alcoholic fatty liver from the perspective of metabolomics, lipidomics, and the gut microbiota. A choline-deficient, l-amino acid-defined (CDAA) diet can interfere with fat metabolism in the liver of mice and fat transport from the liver to peripheral tissues, leading to an excessive fat accumulation in the liver and to the formation of non-alcoholic fatty liver, which is similar to the pathological state in NAFLD patients [10,11]. This model has been widely applied to study the therapeutic effect of drugs on NAFLD [12,13]. In this study, we showed that PPC exerted therapeutic effects on CDAA diet-induced NAFLD mice using liver histopathological evaluation and serum biochemical indicators.

## 2. Results and Discussion

### 2.1. PPC Has Therapeutic Effects on a CDAA Diet-Induced Model of Fatty Liver in Mice

**Figure 1:**
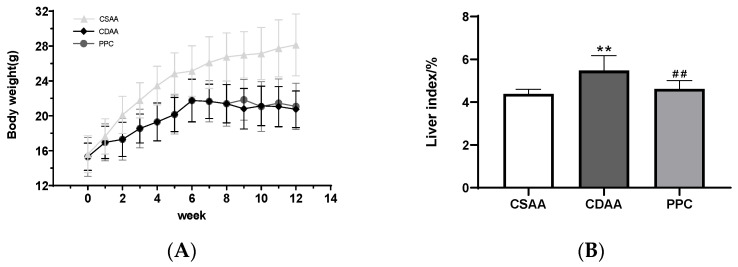
Body weights of mice over 12 weeks (**A**) and the liver index (liver wet weight/body weight ratio) at the end of week 12 (**B**), ** *p* < 0.01 compared with the negative control (CSAA diet) group; ## *p* < 0.01 compared with the disease model (CDAA diet) group.

**Figure 2:**
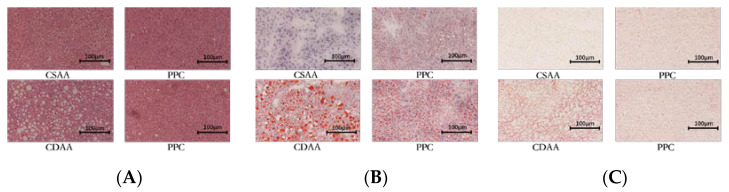
Section with hematoxylin and eosin staining (**A**), section with oil red O staining (**B**), and section with Sirius red staining (**C**) of the mouse liver tissue at 100× magnification.

After being fed a CDAA diet for up to two months in mice, the weight gain was significantly reduced compared with the choline-sufficient, L-amino acid (CSAA) diet group, even for mice undergoing CDAA diet and treated with PPC (Figure 1A). Being an important indicator of fatty liver, the liver index (liver weight/body weight) of the CDAA group was significantly increased, and the liver index of the CSAA group was also significantly smaller than that of the CDAA group after PPC treatment (Figure 1B). The degree of fatty liver lesions was evaluated through histological sections and the NAFLD activity score, as shown in Figure 2 and Table 1.

**Figure 3:**
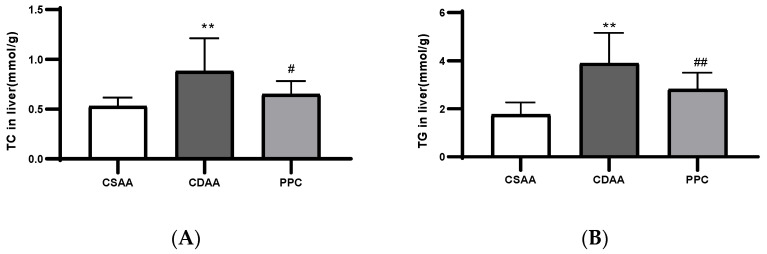
TC (**A**) and TG (**B**) levels in the mouse liver tissues at week 12. ** *p* < 0.01 compared with the negative control (CSAA diet) group; # *p* < 0.05, ## *p* < 0.01 compared with the model (CDAA diet) group.

**Figure 4:**
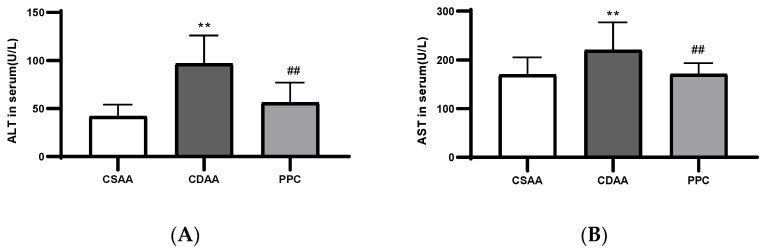

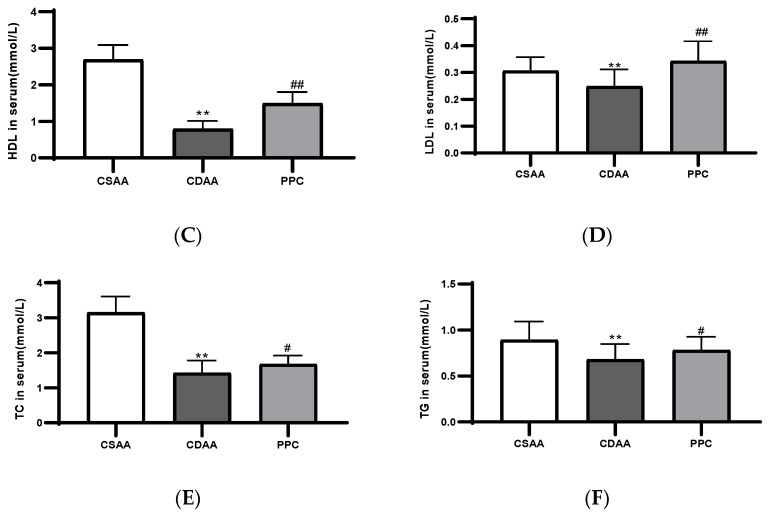
Serum levels of ALT (**A**), AST (**B**), HDL (**C**), LDL (**D**), TC (**E**), and TG (**F**) in the CSAA, CDAA, and PPC groups. ** *p* < 0.01 compared with the negative control (CSAA diet) group; # *p* < 0.05, ## *p* < 0.01 compared with the model (CDAA diet) group.

The accumulation of TC and TG in liver cells is one of the most important features of NAFLD. After a period of disease modeling process through diet, the contents of TG and TC in the liver homogenates in the CDAA group were significantly higher than those in the negative control group, and were significantly reduced after PPC treatment (Figure 3). Serum transaminase concentrations (especially ALT and AST) were also increased in the CDAA diet group, indicating a relatively serious injury to the livers of the mice, whereas the transaminase concentrations were significantly reduced in the PPC-treated CDAA diet group (Figure 4A,B). HDL and LDL levels decreased significantly after disease-modeling and recovered after treatment (Figure 4C,D). A choline-deficient diet could lead to defects in lipoprotein secretion [14], whereas PPC treatment reversed these defects. We found that the serum TG and TC levels of the disease model group were significantly decreased compared with those of the negative control (CSAA), and recovered to some degree after PPC treatment (Figure 4E,F). This could be caused by the disease-modeling principle of choline-deficient feeding: when choline is deficient, the content of PC decreases, and the synthesis and secretion of VLDL in the liver slows down [15], resulting in a reduced rate of lipid transport to the blood and an accumulation of lipids in the liver cells, finally leading to a decrease in the serum lipid level. The same result was demonstrated in the study by Miyaki et al. [16].

The NAFLD model caused by insufficient choline intake includes a number of processes including steatosis, fibrosis, and cirrhosis, which are very similar to the NAFLD development process in humans, and is suitable for studying human NAFLD [17]. Histological examination is still the most accurate method for fatty liver diagnosis. Serum biochemical indicators such as (ALT) are also the most commonly used biochemical markers to assess liver function. After two months of the CDAA diet, all mice in the disease-model group developed severe fatty liver disease, and some mice also developed fibrotic lesions. After one month of PPC administration (150 mg/kg/day), the mice in the treatment group showed significant improvement compared with the model group. These results demonstrate that this dose of PPC has a good therapeutic effect on fatty liver disease in mice. PPC mainly achieves the therapeutic effect from three aspects. Firstly, PPC provides the liver with a large amount of high-energy phospholipids, which penetrate into the liver cell membrane in the form of intact molecules, leading to increased cell membrane fluidity and liver cell regeneration [18]; secondly, it converts static cholesterol into a mobile form, reducing degeneration and necrosis of the liver cells by removing fat from the liver; and thirdly, it enhances the ability of the cell membrane to absorb metabolic cholesterol HDL, and improves the lipid metabolism of the blood and liver [19]. In order to further explore its treatment mechanism, we have evaluated lipidomics, metabolomics, and the gut microbiota.

### 2.2. Effect of PPC on Serum Lipidome

**Figure 5:**
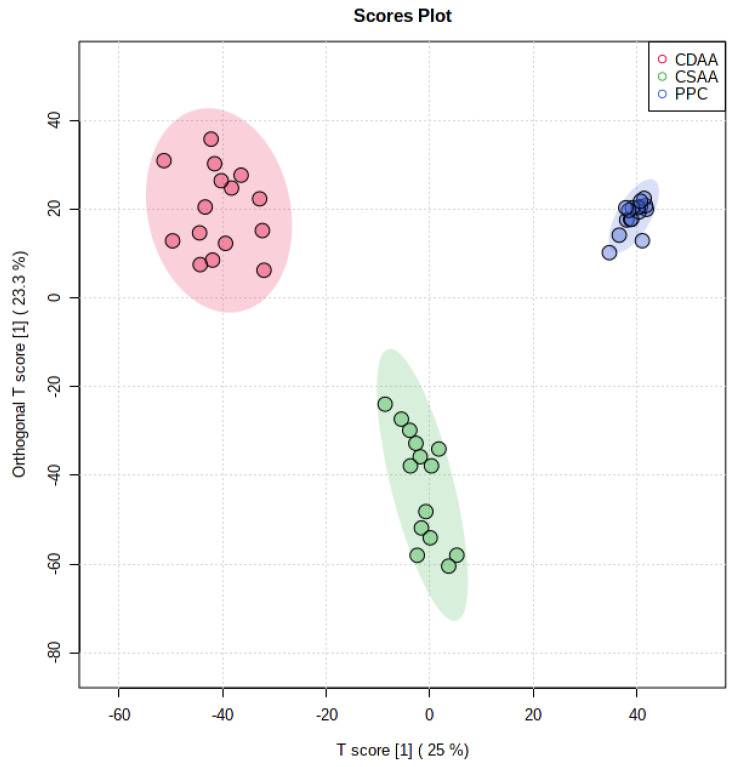
Orthogonal partial least squares discriminant analysis (OPLS-DA) cross-validated classification plot. The OPLS-DA analysis of the groups shows R2X = 0.25, RXY = 0.879, and Q2 = 0.865.

**Figure 6:**
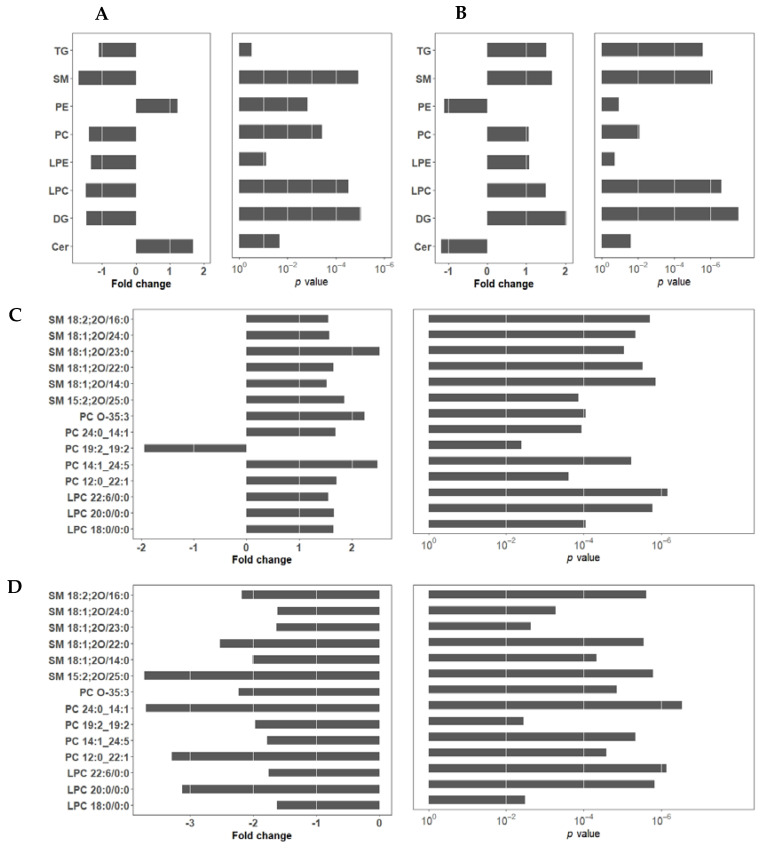
Fold changes in the serum lipid classes before and after CDAA diet feeding (**A**); fold changes in the serum lipid classes before and after PPC treatment (**B**); fold changes in the serum lipid species before and after CDAA diet feeding (**C**); and fold changes in the serum lipid species before and after PPC treatment (**D**).

**Figure 7:**
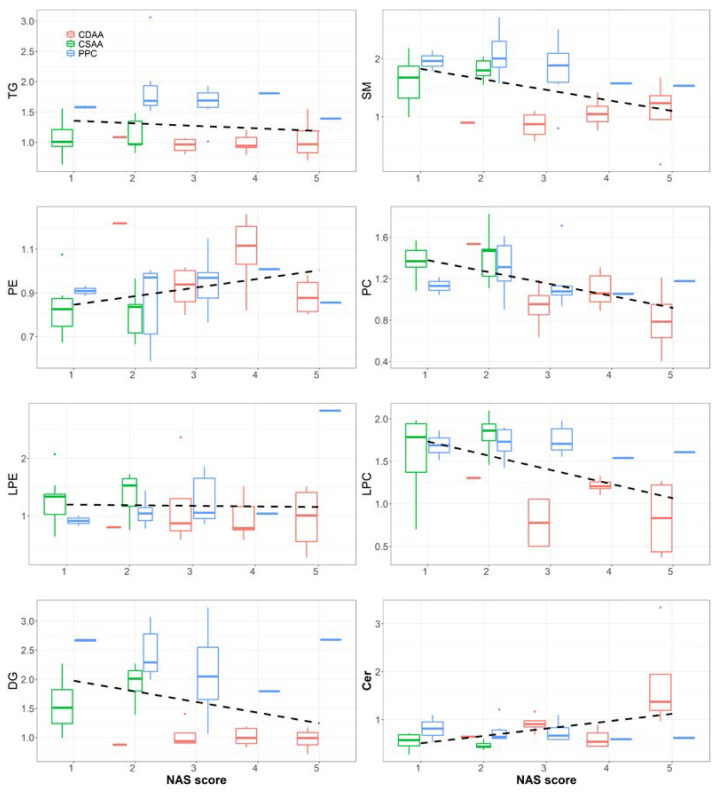
Analysis of the trend between the NAS score and the relative value of the lipids in the serum; a larger NAS value indicates a greater NAFLD severity.

As shown in Figure 5, discriminant analysis exhibited distinct clusters between CSAA and CDAA diet-fed mouse serum samples, indicating considerable variation in the serum lipid composition. PPC-treated samples were well separated from the CDAA group. Further analysis of the lipid abundance of each lipid class revealed that all lipid classes except phosphatidylethanolamines (PE) and ceramide (Cer) were significantly reduced after CDAA diet-feeding (Figure 6A). The level of these lipids in the PPC-treated group showed an opposite effect: all of the lipids except PE and Cer were significantly increased after PPC treatment, compared with the CDAA group (Figure 6B). A reduction in the serum total TG level was associated with corresponding changes in the serum PC and LPC in the same direction. Long-term CDAA feeding was previously reported to lead to a decrease in PC and LPC; the subsequent lack of PC, LPC, and SM led to a decrease in lipoprotein synthesis and secretion, resulting in decreased TG transport from the liver to the serum, an accumulation of TG in the liver and a decrease in TG in the serum [20]. PPC treatment reverses this effect by supplementing PC. The trend analysis between the liver stages and plasma lipids also showed similar results (Figure 7). The NAFLD activity score (NAS) is often used to evaluate the degree of fatty liver disease, and a larger NAS score indicates greater NAFLD severity. The results in Figure 7 show that SM, PC, LPC, and DG were significantly negatively correlated with NAS scores, with a lower SM, PC, LPC, and DG associated with worse liver stages in the CDAA group. PE and Cer showed a contrary tendency, where higher PE and Cer levels in the plasma tended to be accompanied by more severe fatty liver. However, the PPC-treated group tended to have increased levels of LPC, DG, and TG regardless of the NAS stage. Table 2 and Table 3 identified differences in lipid levels (absolute value of fold change > 1.5 and *p* value < 0.05) between the CSAA and CDAA groups, and between the CDAA and PPC groups, respectively. Figure 6C,D shows the lipid species with significant differences before and after disease modeling and treatment. Except for PC 19:2_19:2, the other lipids decreased significantly after disease modeling and recovered after PPC treatment (Figure 6C,D).

### 2.3. Effect of PPC on Serum Metabolome

Table 4 and Table 5 show the absolute value of a fold change > 1.5 (*p* value < 0.05) of the metabolites between the CDAA and CSAA groups, and between the PPC and CDAA groups, respectively. The levels of hexanoylcarnitine, octadecenoylcarnitine, and L-carnitine were significantly decreased in the CDAA group compared with the CSAA group. After treatment with PPC, the abundances of the two acylcarnitines increased significantly, while L-carnitine decreased further. Carnitine is known to be an important biological factor in fatty acid oxidation. It is essential for transporting long-chain fatty acids from the cytoplasm to the mitochondria. Derived from long chain fatty acids and carnitine, acyl-arnitines are transported to the mitochondria by ester linkage. They are converted into acyl CoA on the inner mitochondrial membrane and serve as a substrate for β-oxidation [21]. L-carnitine is reported to have adjuvant therapeutic effects on fatty liver disease and insulin resistance [22,23,24]. PPC has been shown by in vitro experimentations to improve the oxidative substrates of the mitochondria, restore the respiratory chain activity stimulated by ADP, and improve the activity of mitochondrial cytochrome oxidase [25]. Katz et al. reported that PPC can prevent oxidative phosphorylation of the mitochondria, changes in mitochondrial skeleton, loss of mitochondrial cristae, and inhibition of the activities of caspase-3 and caspase-9, thereby inhibiting mitochondrial apoptosis [26]. An association between NAFLD and decreased muscle mitochondrial activity has been reported [27,28]. Pérez-Carreras and colleagues showed that NASH patients presented hepatic mitochondrial abnormalities, which were most likely related to liver fibrosis [29]. Mitochondrial dysfunction is an important factor leading to NAFLD and its disease progression. We hypothesized that PPC could alleviate the appearance of insulin resistance through the protection of the mitochondria, and might reduce the degree of fatty liver disease and delay the progression of further liver fibrosis. PPC could also improve the absorption and utilization of L-carnitine by cells. In the PPC treatment group, the L-carnitine level was further decreased but the acylcarnitine levels increased compared with the CDAA group. PPC and L-carnitine may have a synergistic effect, which needs to be verified by further experimentations.

### 2.4. Effect of PPC on Gut Microbiota

**Figure 8:**
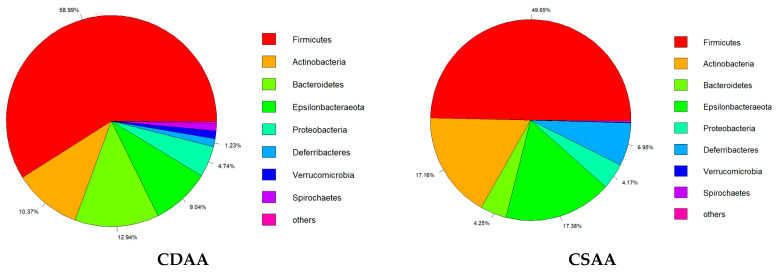

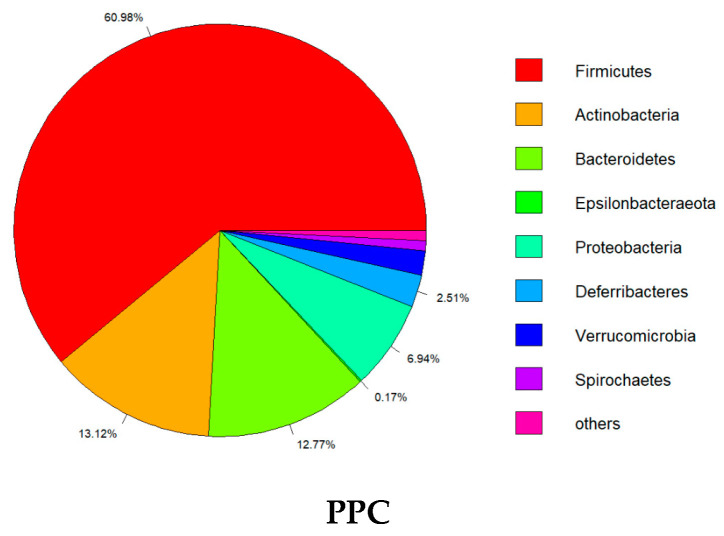
Average phylum distribution of gut microbiomes for CDAA, CSAA, and PPC groups.

**Figure 9:**
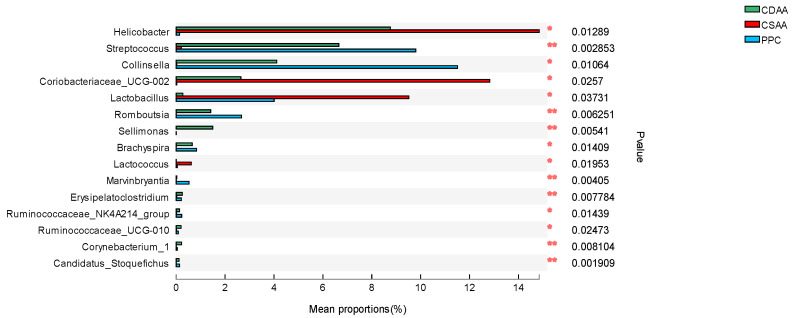
Top ten genuses analyzed using the Kruskal–Wallis test that have significant differences in relative abundance among CSAA, CDAA, and PPC groups, * *p* < 0.05, ** *p* < 0.01.

Abnormal changes in the gut microbiota are closely related to NAFLD [6]. To better explore the therapeutic mechanism of PPC on NAFLD, we conducted a gut microbiota analysis in the mice. Figure 8 shows the overall differences in the gut microbiota of mice in the CSAA, CDAA, and PPC groups. The dominant species of intestinal flora in the three groups of rats were *Firmicutes*, *Actinobacteria*, *Bacteroidetes*, *Epsilonbacteraeota*, *Proteobacteria*, *Deferribacteres*, *Verrucomicrobia*, and *Spirochaetes* (Figure 8). *Firmicutes* was the phylum with the highest abundance in any group. However, compared with the normal CSAA diet, the CDAA diet resulted in a significant increase in *Firmicutes* and *Bacteroidetes*, and the same results were seen in both the CDAA and PPC groups. This suggests that treatment with PPC had no effect on these two phyla. The top ten genuses that have significant differences among the CSAA, CDAA, and PPC groups are shown in Figure 9. The CDAA diet resulted in a significant increase in *Streptococcus*, *Collinsella Romboutsia*, *Sellimonas*, *Brachyspira*, and *Erysipelatoclostridium*, and a significant decrease in *Helicobacter*, *Lactobacillus*, and *Lactococcus*. Only *Sellimona* and *Lactobacillus* were the two species affected by PPC treatment, wherein PPC reversed the effects of the CDAA diet. Studies have found that a significant feature of NAFLD patients on a high-fat diet was an increase in *Firmicutes* and a decrease in *Bacteroidetes*, which could be due to different energy residues in the feces [30,31]. The abnormal bile acid level caused by high-fat diet changed the intestinal pH; *Firmicutes* and *Bacteroidetes* had a different adaptability to the environmental pH [32]. In contrast with using a high-fat diet, we chose choline-deficient diet to develop an NAFLD model. The change in the weight of the mice was opposite to NAFLD patients on a high-fat diet.

Studies have shown that *Lactobacillus* is an important probiotic that is beneficial for maintaining health [33,34]. Jiang et al. isolated two *Lactobacillus* species from Chinese longevity geriatrics, and found that *Lactobacillus* could regulate lipid metabolism in hypercholesterolemic mice models [35]. In a study of 20 adult patients with histology-proven NASH who were randomly allocated to receive a probiotic formula containing *Lactobacillus plantarum*, *Lactobacillus delbrueckii*, *Lactobacillus acido philus*, *Lactobacillus rhamnosus*, and *Bifidobacterium bifidum*, the authors found that patients who had received this formula had a reduced intrahepatic triglyceride content [36]. The development of NAFLD was also associated with the production of alcohol by some intestinal bacteria. A study showed that the blood and respiratory levels of ethanol in NAFLD mice were significantly higher than those in the normal mice, and the activation of AMPK by the *Lactobacillus rhamnoides GG* strain attenuated the accumulation of fat in the liver caused by alcohol [37]. An abundance of *Lactobacillus* may be associated with PPC treatment. The amount of *Lactobacillus* in the CDAA group was significantly reduced compared with the CSAA group; after PPC treatment, *Lactobacillus* was significantly improved. PPC improves NAFLD possibly by multiple mechanisms, one of which is restoring the abundance of *Lactobacilli* in NAFLD mice. Eight weeks of CDAA diet resulted in a significant reduction of *Lactobacilli* in the mouse intestines, while 8 weeks of PPC treatment resulted in a significant recovery of *Lactobacilli*. Whether PPC alleviates fatty liver disease by directly restoring the abundance of *Lactobacilli* or by indirectly leading to an increase in *Lactobacilli* needs further investigation.

## 3. Materials and Methods

### 3.1. Animal Model

Male C57BL/6 mice aged 6 weeks, SPF grade, were purchased from Pengyue Experimental Animal Breeding Co., Ltd., Jinan, China. All healthy male mice were allowed 1 week of acclimatization before the onset of experiments. All mice were randomly divided into two groups: one group (*n* = 18) was fed with choline-sufficient, l-amino acid-defined (Nantong Trophic Feed Technology Co., Ltd., Nantong, China), and another group (*n* = 38) was fed with CDAA (Nantong Trophic Feed Technology Co., Ltd., Nantong, China). After eight weeks of feeding, two mice were selected from the CDAA group to confirm that the model of fatty liver disease was established successfully by liver section. The group of mice fed with CDAA was divided into two groups (CDAA/model and PPC groups with each group consisting of 18 mice), such that the two groups had a comparable average weight. The mice fed with CSAA (negative control group) and the disease model groups were intragastrically fed 10 mL/kg of normal saline every day, and the PPC group was intragastrically fed the same amount of PPC (15 mg/mL, Sanofi Pharmaceutical Co., Ltd., Beijing, China). The mice were weighed once a week and treated with PPC for a total of four weeks. The body weights of the mice in each group were recorded and a two-way analysis of variance was performed to ensure no significant difference between the two groups. At the end of the 8 weeks, the mice were fasted for 12 h and sacrificed. The livers and intestine were rapidly excised and flash frozen in liquid nitrogen. Blood samples were collected and centrifuged at 14,000× *g* rpm for 10 min to obtain the serum samples. All of the serum and tissues samples were stored at −80 °C until analysis. The study protocols were approved by the Institutional Animal Care and Use Committee of Ocean University of China (OUC-AE-2020).

### 3.2. Histological Analysis

The liver tissues of mice were immobilized with 4% paraformaldehyde and embedded in paraffin. The liver was sectioned and stained with hematoxylin and eosin (H&E). To observe the degree of liver fibrosis, liver sections were stained with a picric acid-Sirius red solution. To observe lipid precipitation, the liver tissue was frozen in tissue-Tek OCT (Tissue-Tek, Sakura Finetek, Osaka, Japan) and the sections were stained with oil red O reagent. All of the histological procedures were performed following the standard procedures, as indicated in the reagent specifications. All of the images were captured using an optical microscope (ECLIPSE 80i, Nikon, Tokyo, Japan). Each slice was captured using two different magnifications of light microscopy; NAS scoring was performed on each slice.

### 3.3. Biochemical Indexes Analysis

Commercial kits were used to measure the contents of TG, TC, LDL-C, HDL-C, AST, and ALT (Changchun Huili Biotech Co., Ltd., Changchun, China) in the mice serum according to the manufacturer’s instructions.

### 3.4. Mice Liver Lipid Analysis

A portion of the dissected liver tissues was ground nine times with anhydrous ethanol. After centrifugation at 4000× *g* for 10 min, the supernatants were collected. Commercial kits were used to measure the contents of TG and TC (Nanjing Jiancheng Bioengineering Institute, Nanjing, China) in the liver homogenate of the mice, according to the manufacturer’s instructions.

### 3.5. Lipidomics Analysis

To evaluate the effect of CDAA feeding on the serum lipidome, lipid extracts of the mouse sera were analyzed using a UPLC-Orbitrap mass spectrometer (Thermo Scientific, Waltham, MA, USA). Spectral data were analyzed using MSDIAL (ver 4.24, RIKEN Center for Sustainable Resource Science, Kanagawa, Japan) software, and eight important lipid subclasses were identified [38]. A partial least squares discriminant analysis (PLS-DA) was performed for sample clustering, using the Metaboanalyst 4.0 web portal (www.metaboanalyst.ca, accessed on 20 May 2022). Serum samples were extracted using a method reported previously [39]. A volume of 40 μL serum was mixed with 20 μL internal standard mixture containing lysophosphatidylcholine (LPC) (17:0) and PC (17:0). Then, 800 μL chloroform/methanol (2:1, *v*/*v*) was added and shaken at room temperature for 30 min. The mixture was centrifuged at 14,000× *g* rpm/min at 4 °C for 10 min. The less dense lipid phase was collected and dried under a vacuum at 30 °C. The lipid residue was dissolved in 40 μL isopropanol/acetonitrile (1:1, *v*/*v*). Ultraperformance liquid chromatography–mass spectrometry (UPLC–MS) lipidomic profiling analyses were performed on an Agilent 1290 Infinity UPLC system, equipped with a Waters Acquity UPLC BEH C8 column (2.1 × 50 mm, 1.7 μm particle size; Waters Corporation, Milford, MA, USA), and coupled to an LTQ Orbitrap XL mass spectrometer (Thermo Scientific). Gradient elution was employed in the chromatographic separation method using acetonitrile/water (6:4, *v*/*v*) containing 10 mM ammonium formate and 0.1% formic acid (mobile phase A), and acetonitrile/isopropanol (1:9, *v*/*v*) containing 10 mM ammonium formate and 0.1% formic acid (mobile phase B), with the following program: 0–25 min 32% to 97% B, 25–29 min 97% B, and 29–35 min 32% B. The flow rate was maintained at 0.25 mL/min for 35 min. Both positive and negative ionization mode data were collected, and the mass range was 200–1600 *m*/*z*. MS and MS/MS were collected at a resolution of 70,000 and 17,500, respectively. The electrospray ionization (ESI) conditions were as follows: capillary voltage and temperature were set at 35 V and 300 °C in the positive and negative modes. Quality control (QC) samples prepared from the pooled sera of mice were used to monitor the overall quality of the lipid extraction and mass spectrometry analyses. QC samples were included in the batches of analytical samples during the study. The average coefficient of variation of major lipids detected in the QC samples was <20%. The acquired MS and MS/MS spectral data were analyzed using MSDIAL software for lipid identification according to the instructions in the software tutorial [38,40]. The mass tolerance was set at 10 ppm.

### 3.6. Metabolomics Analysis

A volume of 150 μL serum was mixed with 450 μL methanol and vortexed for 30 s. After centrifugation at 14,000× *g* rpm at 4 °C for 15 min, 500 μL of supernatant was added to a 1.5 mL centrifuge tube. The supernatant was dried under vacuum at 4 °C vacuum. The residue was dissolved in 100 μL acetonitrile/water (1:1, *v*/*v*) and vortexed for 30 s. The samples were centrifuged at 14,000× *g* rpm at 4 °C for 15 min, and the supernatant was collected for analysis. The metabolomics analysis was performed using an Agilent 1290 Infinity UPLC system coupled to a Thermo LTQ Orbitrap mass spectrometer equipped with a heated electrospray ion source (Thermo Scientific, Waltham, MA, USA). Metabolite extracts were separated on a Waters ACQUITY BEH C18 column (2.1 × 50 mm, 1.7 μm) with the column temperature maintained at 40 °C. The mobile phase was water (A) and methanol (B), both containing 0.1% formic acid. The sample was eluted with the following program: 0–1 min 2% B, 1–9 min 2% B to 98% B, 9–12 min 98% B, 12–12.1 min 98% to 2% B, and 12.1–15 min 2% B. The flow rate was 250 μL/min, and the sample injection volume was 8 μL. The mass spectrometer was operated in a positive ionization mode. The full scan was collected at a resolution of 60,000. The data were imported to Progenesis QI (Waters, Milford, MA, USA) software for data processing and analysis. Compounds with a *p* value < 0.05 and fold change value > 1.5 were considered as metabolites with significant change. The MS/MS spectra were compared with those from online databases (HMDB: http://www.hmdb.cal and METLIN: http://metin.scrippsed, accessed on 20 May 2022) for compound identification.

### 3.7. Gut Microbiota

With a sterile scalpel, the entire intestine was taken out in a sterile state. The outer surface of the intestine was cleaned with sterile water. The contents of the intestinal segment up to 3–4 cm of cecum were cut for the intestinal microbial analysis. Microbial genomic DNA was extracted from the intestinal contents of the mice using the E.Z.N.A.^®^ soil DNA Kit (Omega Bio-tek, Norcross, GA, USA), according to the manufacturer’s instructions. The DNA extract was placed on 1% agarose gel; the DNA concentration and purity were determined with NanoDrop 2000 UV–VIS spectrophotometer (Thermo Scientific, Wilmington, DE, USA). The hypervariable region V3–V4 of the bacterial 16S rRNA gene were amplified with primer pairs 338F (5′-ACTCCTACGGGAGGCAGCAG-3′) and 806R 5′-GGACTACHVGGGTWTCTAAT-3′) by an ABI GeneAmp^®^ 9700 PCR thermocycler (ABI, Foster City, CA, USA). The PCR amplification of the 16S rRNA gene was performed as follows: initial denaturation at 95 °C for 3 min, followed by 27 cycles of denaturing at 95 °C for 30 s, annealing at 55 °C for 30 s and extension at 72 °C for 45 s, and single extension at 72 °C for 10 min, and ending at 4 °C. The PCR mixtures contained 4 μL 5 × TransStart FastPfu buffer, 2 μL 2.5 mM dNTPs, 0.8 μL forward primer (5 μM), 0.8 μL reverse primer (5 μM), 0.4 μL TransStart FastPfu DNA Polymerase, 10 ng template DNA, and finally ddH_2_O of up to 20 μL. The PCR reactions were performed in triplicate. The PCR product was extracted from 2% agarose gel and purified using the AxyPrep DNA Gel Extraction Kit (Axygen Biosciences, Union City, CA, USA) according to the manufacturer’s instructions and was quantified using a Quantus™ Fluorometer (Promega, Madison, WI, USA). The purified amplicons were pooled in an equimolar and a paired-end sequenced on an Illumina MiSeq PE300 platform/NovaSeq PE250 platform (Illumina, San Diego, CA, USA) according to the standard protocols by Majorbio Bio-Pharm Technology Co., Ltd. (Shanghai, China). The raw 16S rRNA gene sequencing reads were demultiplexed, quality-filtered by Fastp version 0.20.0 (HaploX Biotechnology Co., Ltd., Shenzhen, China), and merged by FLASH version 1.2.7 (Adobe Systems Incorporated, San Jose, CA, USA) with the following criteria: operational taxonomic units (OTUs) with 97% similarity cutoff were clustered using UPARSE version 7.1 (Shanghai Infinity Biotechnology Co., Ltd., Shanghai, China), and chimeric sequences were identified and removed. The taxonomy of each OTU representative sequence was analyzed using an RDP Classifier version 2.2 (Majorbio Bio-Pharm Technology Co., Ltd., Shanghai, China) against the 16S rRNA database (e.g., Silva v138) using a confidence threshold of 0.7.

## 4. Conclusions

Non-alcoholic fatty liver disease (NAFLD) is a complex disease arising from both genetic and environmental factors. Our study showed that the choline-deficient diet could induce mice to develop severe NAFLD and even NASH. Lipidomics, metabolomics, and gut microbiota analyses combined with histopathological examination and blood routine examination were employed to study the protective effect of PPC against CDAA diet-induced NAFLD mice and its possible mechanisms. The content of major lipids in CDAA diet-induced NAFLD mice was significantly changed compared with that in normal mice, and PPC treatment improved these lipid abnormalities to a certain extent, especially for lipids such as PC, LPC, and SM that are associated with the synthesis of VLDL. Five metabolites were identified to have significant changes before and after disease modeling and treatment. The therapeutic effect of PPC on NAFLD might be related to acylcarnitine metabolism. In addition, the gut microbiota of the three groups of mice also showed significant differences. Further studies are needed to elucidate the mechanism of PPC treatment on NAFLD. Our study evaluated the effect of PPC treatment on an in vivo model of NAFLD, from the perspective of changes in lipidomics, metabolomics, and the gut microbiota.

## Figures and Tables

**Table 1:** NAS score of the CDAA, CSAA, and PPC groups.

	NAS Average Score	Variance
CSAA	1.44	0.60
CDAA	3.78	0.97
PPC-treated CDAA	2.56	0.96

Note: a larger the NAS score indicates a greater NAFLD severity.

**Table 2:** Fold changes of the serum lipid species in the C57BL/6 mice after being fed a CDAA diet.

Lipid ID	*m*/*z*	RT (min)	Chemical Formula	Fold Change *	*p*-Value
LPC 18:0/0:0	524.37	3.17	C_26_H_54_NO_7_P	−1.63	3.21 × 10^−3^
LPC 18:1/0:0	522.36	1.85	C_26_H_52_NO_7_P	−1.66	1.70 × 10^−9^
LPC 20:0/0:0	552.40	4.20	C_28_H_58_NO_7_P	−3.12	1.49 × 10^−9^
LPC 22:0/0:0	580.44	5.31	C_30_H_62_NO_7_P	−2.24	1.88 × 10^−10^
LPC 22:1/0:0	578.42	3.73	C_30_H_60_NO_7_P	−2.40	5.74 × 10^−12^
LPC 22:6/0:0	568.34	1.43	C_30_H_50_NO_7_P	−1.76	5.97 × 10^−9^
LPC 24:0/0:0	608.47	6.78	C_32_H_66_NO_7_P	−2.15	9.27 × 10^−10^
PC 12:0_22:1	760.59	9.14	C_42_H_82_NO_8_P	−3.28	4.18 × 10^−5^
PC 12:0_26:5	808.58	8.45	C_46_H_82_NO_8_P	−2.81	3.94 × 10^−4^
PC 14:0_24:2	814.64	10.34	C_46_H_88_NO_8_P	−3.47	2.26 × 10^−13^
PC 14:1_22:4	780.56	8.50	C_44_H_78_NO_8_P	−2.33	5.37 × 10^−8^
PC 14:1_24:5	806.57	8.25	C_46_H_80_NO_8_P	−1.78	3.94 × 10^−8^
PC 16:0_20:1	788.62	10.01	C_44_H_86_NO_8_P	−2.33	2.04 × 10^−10^
PC 16:0_24:5	836.61	9.98	C_48_H_86_NO_8_P	+5.36	8.29 × 10^−4^
PC 17:1_17:1	758.57	8.73	C_42_H_80_NO_8_P	−3.29	2.77 × 10^−7^
PC 17:2_17:2	754.54	8.36	C_42_H_76_NO_8_P	−3.12	2.00 × 10^−6^
PC 18:2_18:2	782.57	8.40	C_44_H_80_NO_8_P	−3.60	4.26 × 10^−6^
PC 19:2_19:2	810.61	9.56	C_46_H_84_NO_8_P	−1.96	4.74 × 10^−4^
PC 20:2_18:3	808.58	8.78	C_46_H_82_NO_8_P	+1.75	2.54 × 10^−2^
PC 20:3_18:4	804.56	11.68	C_46_H_78_NO_8_P	−3.05	1.91 × 10^−9^
PC 24:0_14:1	816.65	11.68	C_46_H_90_NO_8_P	−3.69	4.29 × 10^−11^
PC O-35:3	756.59	7.92	C_43_H_82_NO_7_P	−2.23	2.12 × 10^−7^
PC O-38:2	800.66	11.11	C_46_H_90_NO_7_P	−1.97	3.88 × 10^−7^
SM 18:1;2O/14:0	675.55	7.91	C_37_H_75_N_2_O_6_P	−2.01	1.12 × 10^−6^
SM 18:2;2O/16:0	701.56	8.08	C_39_H_77_N_2_O_6_P	−2.18	4.61 × 10^−9^
SM 18:1;2O/22:0	787.67	10.77	C_45_H_91_N_2_O_6_P	−2.52	3.60 × 10^−8^
SM 18:1;2O/22:1	785.66	11.58	C_45_H_89_N_2_O_6_P	−3.72	3.66 × 10^−9^
SM 18:1;2O/23:0	801.69	13.39	C_46_H_93_N_2_O_6_P	−1.63	1.15 × 10^−3^
SM 18:1;2O/24:0	815.71	12.39	C_47_H_95_N_2_O_6_P	−1.62	9.68 × 10^−5^

RT, retention time. * The CSAA group is the reference group. “+” refers to an abundance increase in the CDAA group, while “−” refers to an abundance decrease in the CDAA group.

**Table 3:** Fold changes of the serum lipid species in the C57BL/6 mice after PPC treatment.

Lipid ID	*m*/*z*	RT (min)	Chemical Formula	Fold Change *	*p*-Value
LPC 18:0/0:0	524.37	3.17	C_26_H_54_NO_7_P	+1.65	8.89 × 10^−5^
LPC 19:0/0:0	538.39	3.38	C_27_H_56_NO_7_P	+1.91	1.19 × 10^−6^
LPC 20:0/0:0	552.40	4.20	C_28_H_58_NO_7_P	+1.66	1.66 × 10^−6^
LPC 20:4/0:0	544.33	1.74	C_28_H_50_NO_7_P	+2.51	4.60 × 10^−5^
LPC 22:6/0:0	568.34	1.43	C_30_H_50_NO_7_P	+1.55	6.80 × 10^−7^
PC 12:0_22:1	760.59	9.14	C_44_H_82_NO_8_P	+1.71	2.43 × 10^−4^
PC 14:1_20:2	756.56	8.50	C_42_H_78_NO_8_P	+1.76	8.47 × 10^−4^
PC 14:1_24:5	806.57	8.25	C_46_H_78_NO_8_P	+2.49	5.88 × 10^−6^
PC 16:0_22:3	812.62	10.01	C_46_H_86_NO_8_P	−2.51	6.82 × 10^−5^
PC 16:0_24:5	836.62	9.98	C_48_H_86_NO_8_P	−2.18	1.82 × 10^−4^
PC 17:1_17:1	758.57	8.73	C_42_H_80_NO_8_P	−3.96	4.88 × 10^−6^
PC 17:2_17:2	754.54	8.36	C_42_H_76_NO_8_P	+9.50	9.62 × 10^−5^
PC 18:1_18:1	786.60	10.89	C_44_H_84_NO_8_P	−4.16	1.12 × 10^−3^
PC 18:2_18:2	782.58	8.40	C_44_H_80_NO_8_P	+1.75	1.70 × 10^−2^
PC 19:2_19:2	810.61	9.56	C_46_H_84_NO_8_P	−1.93	3.97 × 10^−3^
PC 24:0_14:1	816.65	11.68	C_46_H_90_NO_8_P	+1.69	1.14 × 10^−4^
PC O-35:1	760.62	11.95	C_43_H_86_NO_7_P	+5.26	1.63 × 10^−4^
PC O-35:3	756.59	11.10	C_43_H_82_NO_7_P	+2.24	8.92 × 10^−5^
PC O-36:1	774.64	11.44	C_44_H_88_NO_7_P	+2.17	6.66 × 10^−4^
PC O-37:1	788.65	13.09	C_45_H_90_NO_7_P	+4.43	5.72 × 10^−5^
PC O-37:5	780.58	8.33	C_45_H_82_NO_7_P	+3.31	3.22 × 10^−4^
PC O-38:1	802.67	12.32	C_46_H_92_NO_7_P	+3.15	1.29 × 10^−5^
PC O-38:2	800.66	11.63	C_46_H_90_NO_7_P	+2.20	4.31 × 10^−8^
PC O-39:3	812.66	13.46	C_47_H_90_NO_7_P	+1.57	2.86 × 10^−5^
PC O-39:4	810.62	9.71	C_47_H_88_NO_7_P	−2.97	7.49 × 10^−4^
PC O-40:1	830.70	13.13	C_48_H_96_NO_7_P	+2.97	2.73 × 10^−5^
SM 18:1;2O/14:0	675.55	7.91	C_37_H_75_N_2_O_6_P	+1.52	1.40 × 10^−6^
SM 18:1;2O/16:0	703.58	8.83	C_39_H_79_N_2_O_6_P	+1.51	1.77 × 10^−10^
SM 18:2;2O/16:0	701.56	8.08	C_39_H_77_N_2_O_6_P	+1.56	1.96 × 10^−6^
SM 18:1;2O/22:0	787.67	11.93	C_45_H_91_N_2_O_6_P	+1.65	3.00 × 10^−6^
SM 15:2;2O/25:0	785.66	11.17	C_45_H_89_N_2_O_6_P	+1.86	1.37 × 10^−4^
SM 18:1;2O/23:0	801.69	12.71	C_46_H_93_N_2_O_6_P	+2.53	9.02 × 10^−6^
SM 20:1;2O/21:0	701.56	11.66	C_46_H_93_N_2_O_6_P	+3.29	1.09 × 10^−5^
SM 22:2;2O/19:0	785.63	11.58	C_46_H_91_N_2_O_6_P	+1.60	1.44 × 10^−4^
SM 15:2;2O/26:6	787.67	10.77	C_46_H_79_N_2_O_6_P	+2.59	1.38 × 10^−2^
SM 18:1;2O/24:0	785.66	12.87	C_47_H_95_N_2_O_6_P	+1.57	4.55 × 10^−6^
SM 23:3;2O/20:4	801.69	13.39	C_48_H_85_N_2_O_6_P	+1.77	1.26 × 10^−7^

RT, retention time. * The CDAA group is the reference group. “+” refers to an abundance increase in the PPC group, while “−” refers to an abundance decrease in the PPC group.

**Table 4:** Differences in metabolite levels between CDAA and the CSAA groups.

Metabolite Name	*m*/*z*	RT (min)	Chemical Formula	Fold Change *	*p*-Value
L-Phenylalanine	166.08	2.8	C_9_H_11_NO_2_	+1.7254	0.0019
Hexanoylglycine	174.11	6.7	C_8_H_15_NO_3_	−1.5767	0.0477
L-Carnitine	184.09	0.8	C_7_H_15_NO_3_	−2.3805	0.0006
Tryptophan	227.07	4.5	C_11_H_12_N_2_O_2_	+1.8593	0.0181
Hexanoylcarnitine	260.18	6.3	C_13_H_25_NO_4_	−4.0906	0.0069
Octadecenoylcarnitine	426.35	10.88	C_25_H_47_NO_4_	−1.5700	0.0033

RT, retention time. * The CSAA group is the reference group. “+” refers to an increase in abundance in the CDAA group, while “−” refers to a decrease in abundance in the CDAA group.

**Table 5:** Differences in metabolite levels between the PPC and CDAA groups.

Metabolite Name	*m*/*z*	RT (min)	Chemical Formula	Fold Change *	*p*-Value
L-Phenylalanine	166.08	2.8	C_9_H_11_NO_2_	−1.4643	0.0307
Hexanoylglycine	174.11	6.7	C_8_H_15_NO_3_	+1.8401	0.0002
L-Carnitine	184.09	0.8	C_7_H_15_NO_3_	−2.8127	0.0354
Hexanoylcarnitine	260.18	6.3	C_13_H_25_NO_4_	+2.0028	0.0495
Glu-Ile	261.14	5.3	C_11_H_20_N_2_O_5_	−2.3478	0.0006
21-Deoxycortisol	347.22	8.8	C_21_H_30_O_4_	+2.1645	0.0057
Octadecenoylcarnitine	426.35	10.88	C_25_H_47_NO_4_	+2.0037	0.0001

RT, retention time. * The CDAA group is the reference group. “+” values refer to an increase in abundance increase in the PPC group, while “−” values refer to a decrease in abundance decrease in the PPC group.

## Data Availability

The data that support the findings of this study are available from the corresponding author upon reasonable request.
